# The Changing Disease-Scape in the Third Epidemiological Transition

**DOI:** 10.3390/ijerph7020675

**Published:** 2010-02-24

**Authors:** Kristin Harper, George Armelagos

**Affiliations:** 1 Robert Wood Johnson Health & Society Scholar Program, Columbia University, New York, NY 10032, USA; 2 Department of Anthropology, Emory University, Atlanta, GA 30309, USA; E-Mail: antga@emory.edu

**Keywords:** epidemiological transition, globalization, infectious disease, degenerative disease

## Abstract

The epidemiological transition model describes the changing relationship between humans and their diseases. The first transition occurred with the shift to agriculture about 10,000 YBP, resulting in a pattern of infectious and nutritional diseases still evident today. In the last two centuries, some populations have undergone a second transition, characterized by a decline in infectious disease and rise in degenerative disease. We are now in the throes of a third epidemiological transition, in which a resurgence of familiar infections is accompanied by an array of novel diseases, all of which have the potential to spread rapidly due to globalization.

## 1. Introduction

The epidemiological transition model provides a means for understanding the evolution and spread of emerging diseases [1–3]. As originally formulated by A.R. Omran, it described the major transition in mortality rates observed in high-income countries by partitioning history into three time periods: the “age of pestilence and famine,” “the age of receding pandemics,” and the “age of degenerative and man-made diseases” [4–6]. Since its introduction several decades ago, important modifications to the model have been made, including the delineation of additional major transitions [3]. Omran’s model has attracted the attention of demographers [7–9], medical anthropologists [10,11], economists [12], and public health policy workers [13–15]. To date it has received scant attention from epidemiologists, however [16]. While the epidemiological transition model has been discussed in some epidemiological journals [17–21], overall this framework for understanding the prevalence of different types of diseases has had little impact in the field.

Why might this be? In many of the disciplines mentioned above, in which the epidemiological transition model has been most influential, investigators are primarily concerned with health writ large or all-cause mortality rather than individual diseases. Therefore, a model that helps explain a major transition in the human disease-scape is viewed as useful, providing insight into ultimate causes and possible solutions aimed at improving health. In contrast, in epidemiology investigators are often concerned with one disease at a time. The identification of a novel pathogen or the characterization of a new disease requires epidemiologists to pinpoint, as quickly as possible, the properties specific to it: risk factors, life cycle, ecology, vectors, treatments, *etc.* Thus, in epidemiological studies emerging diseases are usually conceptualized as individual entities that can be attributed to particular proximate causes, rather than members of a suite of diseases that are increasing simultaneously due to common, ultimate causes. Though it is certainly important to recognize and describe the particulars of a given pathogen, we argue in this paper that synthesizing such data using the epidemiological transition theory is likely to prove useful to epidemiologists as well. Situating a disease within a particular context using the transition model may provide clues to possible proximate as well as ultimate causes, prevention strategies, and predictions regarding future trends.

In this article, we describe past and present epidemiological transitions. In addition to considering evolutionary factors in the emergence of disease, we will incorporate the spread of drug resistance and explore the implications of globalization in expanding the threat posed by some diseases. We also discuss how the epidemiological model allows us to examine the recent rise of certain classes of disease, such as the frequency of allergies and autoimmune diseases, in high-income countries. Finally, we discuss the ways in which increased attention from epidemiologists may clarify and improve the model.

## 2. Background

Omran [4] developed the concept of the original “epidemiological transition” to describe the shift from mortality due to acute infectious diseases to death via chronic, non-infectious, degenerative diseases. This shift occurred as a result of higher standard of livings and the introduction of medical and public health practices in high-income nations undergoing industrialization. This epidemiologic transition, according to Omran, occurred in three successive phases, as described above, and took about two centuries until completion in Western Europe and the United States. Omran’s model focused on a major change in the pattern of health and disease and explored how these patterns were influenced by demographic, economic, and sociological factors.

**Figure 1.**
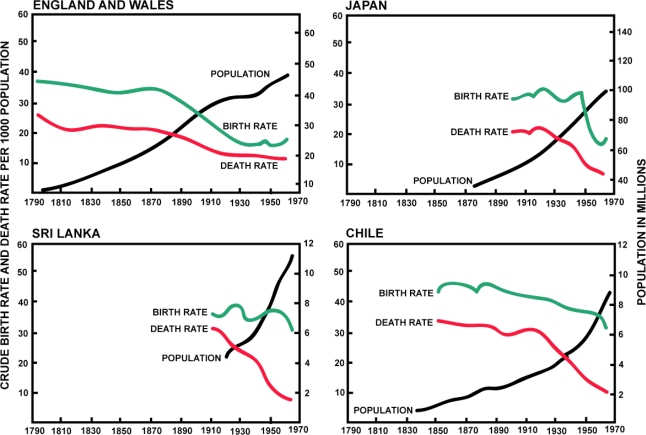
Birth rates, death rates, and population size over the last two centuries in four different areas, illustrating the demographic changes that prompted the development of the epidemiological transition model. Modified from Omran [4] with permission to reprint from John Wiley and Sons.

Epidemiological transition theory is derived from demographic transition theory. Demographic transition theory, first proposed by Thompson [22] and expanded by Notestein [23] and Davis [24], is a generalized model of population structure that is the basis for understanding fertility and mortality processes in contemporary populations (Figure 1). In the first stage, thought to represent most of human evolutionary history, populations experience near maximal fertility and mortality rates, resulting in little increase in population size. In the second stage, populations experience a decrease in mortality rates while fertility rates remain high, resulting in a rapid increase in population size. In the third stage, mortality rates are low and birth rates begin to decline, resulting in slowed population growth. In the last stage, low mortality and fertility rates result in no increase in population size. Epidemiologic transition theory explains an important component of the demographic transitions by describing the varying patterns of disease that are important contributors to mortality.

As the model of epidemiologic transition became increasingly popular and drew more attention, modifications to it began. Both the demographic transition and Omran’s epidemiological transition theories assumed that the earliest human populations were rife with disease, resulting in communities in which extremely high mortality was compensated for by maximum fertility. For example, Omran’s “age of pestilence and famine” assumed that life in the Paleolithic period was disease-ridden, with food in short supply. This portrayal reflects the popular conception of prehistoric life as “nasty, brutish, and short”. But was this actually the case? Evidence to the contrary has existed for some time. In the Eastern Mediterranean, studies of skeletal samples demonstrate that the post-Paleolithic shift to agriculture was accompanied by progressively worse nutrition, as evidenced by a decrease in stature, and an increase in disease, as indicated by porotic hyperostosis and enamel hypoplasias [25]. Similar results were found in those New World populations in which maize was introduced and became an increasingly important source of food [26,27]. This has led some researchers to believe that rather than being limited by high mortality rates, Paleolithic population size may have been kept in check by low fertility rates [28].

In addition, researchers began to see the need for a model that included additional epidemiologic transitions. Rather than Omran’s single transition, an expanded model including three transitions has been described [2,3,29]. In this model, the first transition is one that predates that described by Omran: it marks the shift from foraging to agriculture. Prior to the advent of agriculture, our Paleolithic ancestors would have encountered novel patterns of disease as they moved into new ecological niches [30–33]. However, their mobility, small population size, and low density would have precluded most modern infectious diseases from representing major selective pressures in such groups [34–36]. With the shift in subsistence, there was a dramatic increase in infectious disease that resulted from an increase in population size and density, the domestication of animals that served as disease reservoirs, increased exposure to disease vectors, and sedentary lifestyle [37,38]. The transition to primary food production was so dramatic that it has been called the Neolithic revolution, and it was marked by the appearance of social stratification that created a significant differential in the risk of disease within a community [29,39,40]. The increase in disease, and thus mortality, that marks the first epidemiological transition accelerates as agriculture intensifies in an area and spreads to new regions.

In the expanded model, the second epidemiological transition represents that described by Omran. It was a period in which the development of improved nutrition and standard of living, public health measures, and, possibly, novel medical treatments resulted in a major decline in infectious disease and mortality rates. As more and more of the population reached advanced age, thanks to the reduced infection threat, a rise in chronic diseases that typically began later in life became evident.

The last transition in this model, the third, is the one that we are entering now. It is characterized by the continued prominence of chronic, non-infectious disease now augmented by the re-emergence of infectious diseases. Many of these infections were once thought to be under control but are now antibiotic resistant, while a number of “new” diseases are also rapidly emerging. The existence of pathogens that are resistant to multiple antibiotics, some of which are virtually untreatable, portends the possibility that we are living in the dusk of the antibiotic era. During our lifetime, it is possible that many pathogens that are resistant to all antibiotics will appear. Finally, the third epidemiological transition is characterized by a transportation system that results in rapid and extensive pathogen transmission.

## 3. Improvements upon Earlier Models

One advantage of the epidemiological transition model is its scope. It has been noted that early epidemiological models that considered the host, pathogen and environment were narrowly focused [41]. They focused on basic environmental variables such as climate, temperature, moisture and the availability of nutrients that affected pathogen growth, but they failed to consider broader political and economic factors that affect disease risk and progression. Meredith Turshen [42] describes these ecological model as “bourgeois” because of their failure to incorporate economic factors such as poverty and inequality, which bring individuals into the path of disease vectors. In this view, these models focus on the proximate cause of disease (the pathogen) without considering the ultimate causes of disease: sociocultural factors [43]. For example, such models fail to consider the way in which large-scale social forces influence economically disadvantaged individuals [44]. The importance of economic disparities is illustrated through a comparison of health statistics from the Americas and Africa. Combined, North and South America employ 37% of the world’s health workers, spend more than 50% of the money expended on health worldwide, and host 10% of the global burden of disease. In contrast, Africa has only 3% of the world’s health workers, spends only 1% of the world’s health expenditure, and bears 24% of the world’s disease burden [45: Xviii–XiX].

The epidemiologic transition model is easily expanded to accommodate a broad consideration of the causes of disease and their impact on a population’s ability to adapt in various ways [46]. On the one hand, the array of proximate causes of disease considered is more comprehensive. Following the lead of Audy and Dunn [47], there has been a shift to consider a broader category of insults as the source of disease. Insults now include all factors which adversely affect the ability of the host population to respond to the environment successfully, including pathogens [48,49], toxins [50,51], physical forces which cause trauma [52], pollutants [53], and even psychological factors [54]. On the other hand, the utility of the model for understanding the ultimate causes of disease is facilitated by a focus on the population as the unit of study rather than the individual. This modification represents an important change in perspective that resulted from the influence of population biology [55,56] on human disease ecology. Using the population as the unit of study allows us to move beyond the clinical perspective in order to consider diseases in their broader biological and social context [57].

Jacque May [41], a proponent of such comprehensive models, provides an example illustrating the importance of considering the cultural system as part of human-disease interaction. It had been noted that despite living in a setting ideal for the transmission of malaria, highland Vietnamese seemed to be immune to the disease. This situation was difficult to explain using traditional ecological models. However, May noticed that they built their houses on stilts, *nha san*, that were six feet above ground, with livestock maintained under the houses. All day, cooking fires were kept burning in their homes. This set-up proved ideal for keeping malaria-carrying mosquitoes out of their living quarters. *Anopheles minimus*, the malaria vector in this area, has a flight pattern of about three feet above ground and, thanks to the stilts, contents itself with feeding on the livestock kept below the homes. When mosquitoes do stray above the usual three feet, the smoke in the house serves as a further deterrent to keep them away from its inhabitants [58].

The example of the highland Vietnamese scenario demonstrates how culture can act as a buffer to disease transmission, shielding the population from insults that originate in the environment. There are, however, instances in which cultural systems can themselves produce insults. Returning to the Vietnam scenario, May [41] described how the lowland *Kinh* populations feared malaria when resettled in upland areas; this fear proved legitimate in light of the high prevalence of the disease among them. Why were the *Kinh* so susceptible? The *Kinh* belief system attributed malaria to malevolent spirits [58]. Unlike the highland dwellers, they built their homes on the ground, had kitchens detached from their houses, and corralled their livestock away from living areas. This pattern of habitation put the *Kinh* in the path of low flying *Anopheles* without protective smoke or livestock that provided alternative meals for the mosquitoes. In the years since May’s study, the *Kinh* have successfully adapted to the area by first understanding that mosquitoes are the source of malaria and second taking appropriate technological means to protect themselves. They now employ bed nets, take anti-malarial drugs and dam streams in order to create a more healthful environment [58].

## 4. Profiles of the Major Transitions

Here, we discuss each of the time-periods in the three major transitions outlined above in more detail (Table 1).

### 4.1. The Paleolithic Baseline

In order to understand the dramatic shift that occurred with the transformation to primary food production, it is necessary to reconstruct the Paleolithic pattern of diet and disease as a baseline. The archeological record provides direct, if incomplete, evidence of patterns of adaptation in foraging hominine populations. Analyses of stable isotopes [59], the remains of meals [60], and facial architecture [61] provide clues to the early hominid diet [62]. It appears that Paleolithic foragers tended to eat a varied diet, including different relative amounts of terrestrial mammals, fish, shellfish, birds and plants based on the resources available in a given environment [63]. The Pleistocene disease profile is even more difficult to reconstruct than diet. There are relatively few skeletal remains from this period, and even fewer bear osseous signatures of disease. For this reason, we have to rely primarily on theoretical reconstructions based on our knowledge of hominine habitat, the pattern of disease found in contemporary forager populations, and genomic analyses of humans and their pathogens.

The early hominid environment has been the subject of some controversy. Early views of human evolution suggested that bipedalism was a development that allowed our ancestors to adapt to the savannah [64,65], which has been assumed to be the human homeland. However, recent geochemical [66] and flora and faunal analyses [67–71] argue for a woodland or grassy woodland hominine origin. Which habitat represents the environment from which we emerged has important implications for understanding our earliest disease profile, since that of a tropical grassy woodland would be quite different from that of a savannah environment. Inhabitants of a grassy woodland, for example, may have been more likely to encounter the *Anopheles* mosquito that transmits malaria.

The disease ecology of contemporary hunter-gatherers also provides a model for that of Paleolithic foragers. For example, from studies of contemporary groups we understand that the population size reached by foraging groups would be too small to support continuous transmission of many of the “crowd” diseases. For example, infections such as influenza, measles, mumps, and smallpox require too large a host population to have been present in the Paleolithic disease-scape [72]. We can also speculate that these foragers would have been exposed to the many saprophytic mycobacteria that were present in the soil and decaying organic matter. Stig Bengmark [73, p. 612] has compared the modern Western diet with that which sustained Paleolithic populations. He estimates that our Paleolithic ancestor’s diet contained at least a billion times more non-pathogenic bacteria, primarily of the *Lactobacillus* variety, that can promote health. Many cultures continued to rely on fermentation until recently, resulting in supplementation of the diet by these bacteria; however, in Western countries consumption of *Lactobacillus* has fallen dramatically in the last century. Bengmark asserts that humans in rural, low-income settings have a health-protecting commensal flora that weighs 2 kg., on average, as compared to a weight of 1.3 kg. in the residents of high-income counties. Thus, studies of modern foragers suggest that epidemiological transitions involve health-promoting as well as health-destroying microorganisms.

Finally, the genomic diversity of pathogens and parasites provide clues to their phylogenetic relationships and patterns of adaptation to their hosts. Armelagos and Harper [74,75] analyzed the genomic patterns in domesticates, pathogens, and humans to understand factors at work in the origin of agriculture. It is sometimes possible to use the molecular clock to estimate when a pathogen began to parasitize its host, and some results from genetic analyses have been surprising. For example, it was long assumed that tapeworms emerged as human parasites during the Neolithic, when new proximity to domestic cattle and pigs would have provided opportunity for transmission. Molecular analyses by Eric Hoberg [76,77], however, suggest that the human parasite sister species (*T. saginata* and *T. asiatica*) differentiated about 160,000 years ago, around the time that humans migrated out of Africa. The data suggest that hominids and carnivores, such as big cats, preyed on similar grazing animals; it was from their common prey that humans initially became infected with tapeworms. Subsequently, humans may have actually transmitted the tapeworms to domesticated cattle and swine, rather than the other way round.

The pathogens that would have afflicted gatherer-hunters can be divided into two classes. “Heirloom species” infected our anthropoid and hominine ancestors and continued to parasitize them as they evolved into hominids. Head and body lice (*Pediculus humanus*) and pinworms (*Enterobius vermiculari*) are examples of heirloom species. Lice have been ectoparasites since the Oligocene, afflicting a variety of species since then [78]; it appears that the species of lice specific to humans traveled with us out of Africa, diverging into head and body lice on multiple occasions [79]. Other heirloom species probably include most of the internal protozoa found in modern humans, as well as such bacteria as *Salmonella typhi* and staphylococci [34,80]. It is possible that yaws, a non-sexually transmitted disease caused by a bacterium related to the one that causes syphilis, is also an heirloom disease; it infects our closest relatives, chimpanzees and gorillas [81,82], and genetic analyses indicate that it has an ancient origin in humans [83,84].

In contrast to heirloom species, “souvenir” species are those that are “picked up” along the way, as hominids carry out their daily activities. Souvenir species are usually zoonotic pathogens whose primary hosts are non-human animals; they infect humans only incidentally. Zoonoses are passed on to humans through insect and animal bites, preparing and consuming contaminated flesh, and via contact with urine and feces of infected animals. Sleeping sickness (trypanosomiasis), tetanus caused by the toxin produced by *Clostridium tetani,* scrub typhus (*Orientia tsutsugamushi*), relapsing fever caused the spirochete *Borrelia*, trichinosis from the roundworm *Trichinella spiralis*, tularemia (*Francisella tularensis*), avian or ichthyic tuberculosis, leptospirosis (from the spirochete *Leptospira* spp.), and schistosomiasis are among the zoonotic diseases that likely afflicted earlier gatherer-hunters [35]. As discussed above, small population size would have precluded sustained transmission of many bacteria and viruses, though Cockburn [80] has argued that some viral diseases found in non-human primates could have been easily transmitted to early hominids. Malaria may also fall into this category. If malaria was contracted by humans in the Pleistocene, it likely would have been in isolated incidences. For example, recent genetic analysis of the glucose-6-phosphate dehydrogenase gene, some variants of which confer resistance to the infection, confirmed that malaria is a recent selective force in human populations, occurring within the last 10,000 years [85]. Based on the mitochondrial genome of the parasite itself, Joy *et al.* concluded that though the parasite that causes falciparum malaria originated long ago (perhaps 50,000–100,000 YBP), a sudden increase in the population size of the parasite did not occur until around 10,000 years ago [86] when humans began to practice agriculture.

### 4.2. The First Epidemiological Transition

The disease-scape changed dramatically after the adoption of agriculture. New proximity to domestic animals created many opportunities for novel pathogens to infect, and eventually adapt, to humans. It has long been thought that many of our most feared diseases (anthrax, tuberculosis, Q fever, brucellosis, smallpox, measles, *etc.*) emerged at this time, evolving from progenitors contracted from goats, sheep, cattle, pigs, and fowl. Not all of these origin stories have held up under closer scrutiny. For example, analysis of the *Mycobacterium tuberculosis* genome rules out linear evolution of the human pathogen from *M. bovis*, the species that infects cattle [87] and suggests that the former pathogen may actually have appeared prior to the latter, and not vice-versa [88]. Nevertheless, it is clear that many important human infections did initially arise from close contact with domestic animals. Peri-domestic animals such as rodents and sparrows, which developed permanent habitats in and around human dwellings, could also represent important sources of disease, such as the bubonic plague, hantavirus, typhus, *Salmonella*, and histoplasmosis.

The very act of farming may have resulted in exposure to novel pathogens as well as increasing the risk of contracting familiar infections. The cultivation of soil, which requires the breaking up of sod, may have exposed farmers to the chiggers that carry the bacterium *Orientia tsutsugamushi*, the causative agent of scrub typhus [89]. Similarly, Livingstone [90] argued that slash-and-burn agriculture in West Africa would have exposed populations to *Anopheles gambiae*, the mosquito that serves as the vector for *Plasmodium falciparum*, the cause of malaria. Slash-and-burn agriculture resulted in sedentary populations surrounded by the pools of sunlit water required for propagation of the *Anophelese* mosquito. *Aedes aegypti*, the vector that carries yellow and dengue fever, breeds in artificial containers; frequent contact with this mosquito is also likely to have begun and intensified around the time that sedentary settlements became common. Finally, agricultural practices such as irrigation and the use of human feces as fertilizer would have increased exposure to pathogens such as the one that causes schistosomiasis [35].

Changes in nutrition and food handling would also have altered disease risk. The shift to agriculture resulted in a reduction of the dietary niche, which would have predisposed many individuals to dietary deficiencies uncommon in the Pleistocene. For example, porotic hyperostosis, a skeletal marker indicative of anemia (including that caused by iron-deficiency) first appears in the Upper Paleolithic, increasing throughout the Neolithic [91]. Nutritional deficiencies, which alone were sufficient to cause disease, would also have altered host immune competence, making humans in this time-period more susceptible to infection following contact with a pathogen [92]. Agriculture also resulted in regular food surpluses that had to be stored in large quantities and widely distributed, which probably resulted in outbreaks of food poisoning [93].

In sum, Cohen and Armelagos [94] provide a number of case studies that show a decline in health following the Neolithic transformation, suggesting that this period in human history (a period with different start and end points in different areas) could indeed be regarded justifiably as an age of pestilence and famine. The increasing class inequalities, epidemic diseases, and dietary insufficiencies would also have added mental stress to the list of illnesses that plagued agriculturalists.

### 4.3. The Second Epidemiological Transition

In the last 200 years, a number of developed nations have undergone a second epidemiological transition. This second epidemiological transition represents the original disease transition described by Omran. It was a period in which the development of medical practices, improved nutrition, and public health measures resulted in a decline in early mortality resulting from infectious disease [8,95–98]. Life expectancy at birth in the United States, for example, increased from 49 years in 1900 to 74 years in 1980 [99]. As populations aged thanks to the reduced infectious disease threat, they began to experience a concomitant rise in chronic disease. Deaths caused by chronic conditions such as heart failure, cancer, and diabetes became much more common during this time period; as more and more of the world experiences this transition, the burden of mortality and morbidity increasingly shifts to such causes.

The source of the second epidemiological transition remains controversial. Some have argued that it was an outgrowth of developments in technology, medicine, and science: in particular the germ theory of disease. This hypothesis, however, is one source of contention [100]. While there was a better understanding of the cause of infectious disease at this time and this admittedly resulted in increasing control over many contagious diseases, McKeown argued that the role of medicine in the decline of mortality was minimal [100]. He showed that the morbidity and mortality caused by diseases such as tuberculosis and diptheria declined long before effective medical interventions, such as antibiotics and immunizations, were developed and implemented. Nevertheless, in recent years such medical interventions have resulted in important public health victories, such as the eradication of smallpox. Critics of McKeown have focused on his use of evidence for improved nutrition [101–103] and failure to consider improvements in public health practices [101–105]; however, his thesis of the primacy of nutrition and standards of living in improved health continues to enjoy popularity [106–110].

In addition to an increase in the chronic disorders listed above, many researchers have noted an apparent increase in allergies and diseases of the immune system in the last half of the 20th century [111,112]. The hygiene hypothesis argues that in high-income nations the lack of childhood exposure to infectious pathogens and symbiotic microorganisms increases susceptibility to allergy and similar diseases caused by alterations in the immune system [113]. The theory holds that during the Paleolithic, the impact of infectious diseases was rather minimal but foragers were exposed to many saprophytic microorganisms present in the soil and in decaying plant matter. After the shift to agriculture spurred the first epidemiological transition, exposure to harmless environmental organisms and helminths continued and was augmented by exposure to additional infectious agents. Now that some populations have undergone the second epidemiological transition, better control of infectious diseases as well as the development of a sanitized water supply and sewer system has dramatically decreased contact with microorganisms of all types.

Early versions of the hygiene hypothesis tended to focus on the importance of childhood infections [114]. However, recently Bremner and coworkers [115] have shown that exposure to childhood infections does not appear to protect against allergies in later life by investigating the relationship between infections during infancy and the later development of hay fever in two UK birth cohorts. In response to findings such as these, it has been hypothesized that rather than exposure to the traditional suite of childhood infections, it may be early exposure to commensal bacteria, helminthic parasites, and “pseudo-commensals,” organisms that do not replicate in the human gut but are always present there, that protects against autoimmune disease in adulthood [116–120]. Thus, a modified hygiene hypothesis (‘The Old Friends Hypothesis’ proposed by G.A. Rook) excludes childhood diseases as a requisite factor and focuses instead on the saprophytic micro-organisms and helminthic parasites that are tolerated by the immune system and are absent from the microbe load of high-income nations. Exposure to these ubiquitous agents is postulated to aid in the development of a healthy T regulatory response; lack of early exposure may result in the later manifestation of allergies and an array of autoimmune diseases such as inflammatory bowel disease, multiple sclerosis, and Type 1 diabetes.

Improvements in hygiene and sanitation, as well as the availability of antibiotics to treat many bacterial infections, may have also resulted in a relative increase in the disease burden represented by sexually transmitted infections. Researchers have noticed that some STIs form “disease pairs” with closely related, non-sexually transmitted pathogens [121,122]; examples include HSV-1 (oral) and 2 (genital), ocular and genital Chlamydia serovars, and syphilis and yaws. When the non-sexually transmitted partner decreases in prevalence, there may be more susceptible individuals in the population for its partner to infect, if cross-immunity between the two exists. This could help explain the concomitant decrease in the prevalence of HSV-1 and increase in HSV-2 infections in high-income countries [123–128]; it has similarly been observed that when yaws incidence decreased as a result of eradication programs or increased hygiene, syphilis infection rates rose [129,130]. While the decline in competitor-infections may serve as a partial explanation for the prominence of STIs in the communicable disease burden of high-income countries, the stigma associated with seeking treatment as well as the difficulty in making the type of behavior changes that reduce risk must certainly also play a role. For example, despite the ready availability of antibiotics in the United States during the years 1950−1980, gonorrhea infections rose more than three-fold [131], resulting in five times more cases of this STI than of chickenpox in the year 1979. Thus, STIs as a group remained relatively impervious to the epidemiological transition that reduced other communicable infections in high-income countries.

### 4.4. The Third Epidemiological Transition

We are now entering a third epidemiological transition, one characterized by the re-emergence of infectious diseases previously thought to under control [132–134] as well as the rapid emergence of a number of “new” diseases. The existence of antibiotic resistant pathogens, including those resistant to virtually every agent available [135,136], portends the possibility that we are living at the end of the antibiotic era. These dramatic changes are driven by a transportation system that globalizes the disease process [137], with the transmission of pathogens so rapid and so extensive that we are now said to be connected by a virtual “viral superhighway” [138].

The emergence and reemergence of infectious diseases has been one of the most interesting evolutionary stories of the last decade and has captured the interest of scientists and the public. Satcher [139] and Lederberg [140] list almost twenty-nine diseases that have emerged in the last 28 years, many of them zoonoses. Morse [141] views emerging infections as a result of demographic and technological changes, international commerce and travel, and the breakdown of public health measures and microbial adaptation. Among the ecological changes he describes are agricultural development projects, dams, deforestation, floods, droughts and climatic changes that have resulted in the emergence of diseases such as Argentine hemorrhagic fever, Korean hemorrhagic fever (Hantaan) and Hantavirus pulmonary syndrome.

It should be noted that the focus on emerging diseases in scientific literature and the media has been criticized by some. Paul Farmer [44,142] has asserted that we “discover” emerging diseases when they enter the consciousness of the wealthy [44]. For example, Farmer has argued that hemorrhagic fevers, including Ebola, were described long ago and that, in many cases, their etiologic agents were identified decades ago. Still other diseases grouped under the “emerging” rubric are ancient and well-known foes that have somehow changed, either in pathogenicity or distribution. Multidrug-resistant tuberculosis and invasive or necrotizing Group A streptococcal infections are cases in point. It is only when these diseases enter the consciousness of the wealthy that they are considered a credible threat and designated “emerging” or “re-emerging.” This may occur because the powerful begin to be affected (as in recent decades, when Lyme disease began to increasingly infect the residents of affluent East Coast suburbs) or because an infection captures the popular imagination (as happened with Ebola after the movie Outbreak and the book *The Hot Zone*). For this reason, Farmer emphasizes that even the more holistic of ecological perspectives, those that consider human behavior as well as microbial changes in seeking to understand the emergence of novel pathogens, fail to place the process of disease emergence in a political-economic context.

**Figure 2.**
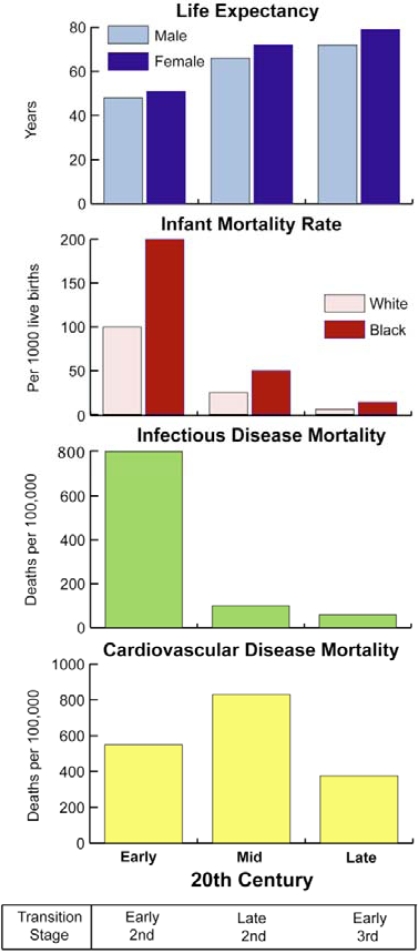
Changes in mortality patterns in the 20th century United States [Based on data drawn from 147–150].

It has also been observed that those of us in high-income countries appear to be entering an era of delayed chronic diseases (Figure 2). In the United States, the incidence of heart disease declined by more than 25% between the years 1968 and 1978 [99]. The death rate from other degenerative diseases, such as stroke and cancer, has also declined since the early 1970s. Olshansky and Ault have argued that this shift is caused by disease onset occurring at increasingly older ages. However, recently it has been predicted that the increase in life expectancy resulting from the era of delayed chronic diseases may not last; increasing rates of obesity, and the diseases associated with it, may lead to a decrease in life expectancy in the United States, and other high income countries, during the 21st century [143].

Globalization is not a new phenomenon, but it is one that is continuously intensifying. William McNeal has described its centuries-old roots and its prominent role in driving change in the global disease-scape [144]. Perhaps the most dramatic disease consequence of early globalization occurred after European explorers initiated contact with the Americas; it is estimated that millions of Native Americans died of diseases such as measles and smallpox as a result. Today, dramatic examples of the spread of disease linked to globalization abound. Often, examples of infectious diseases crossing the Atlantic garner the most attention. For example, SARS spread rapidly from its epicenter in East Asia to North America, as ill passengers went about their travel plans. Many of the most important results of globalization, with regard to disease burden, attract much less notice however. For example, lung cancer rates are increasing in many low-income countries just as they are decreasing in many high-income countries, as cigarette manufacturers in the latter nations shift their marketing focus [145]. Similarly, the export of the Western diet has resulted in a diabetes epidemic that stretches across the world [146].

## 5. Challenges to the Epidemiological Transitions Model

Although the picture of epidemiological transitions presented thus far appears rather clear, in reality the model must be quite complex in order to accommodate observations made in different nations. Transitions can occur at dramatically different paces in different places. Omran’s “classic” model was developed based on findings in the U.S. and Western Europe; he estimated that it took about 200 years, from start to finish, though other authors have argued that it actually began even earlier [151,152]. In contrast, observers have noted that in countries such as Japan and those of Eastern Europe, in which the second transition began later, progress has been much faster [16]. It has also become clear that in many places the transitions are not distinct and sequential. Many low-income countries that never truly benefited from the second epidemiological transition, such as Guatemala, El Salvador, Kenya, and the Democratic Republic of Congo, are already suffering from the consequences of the third epidemiological transition, as infections become antibiotic resistant [153–155]. In the many countries marked by stark social inequalities, diseases associated with under-nutrition and over-nutrition (or the first and second epidemiological transitions, respectively) often co-exist; surprisingly, this combination of underweight and overweight occurs at high levels even within the same family in countries such as China (23%), Brazil (44%), and Russia (57%) [156].

## 6. Conclusion

In this article, we hope to have made it clear how the epidemiological transition model can prove useful to epidemiologists. It provides a means through which findings on individual diseases in disparate populations can be synthesized into a framework that facilitates understanding and intervention. As globalization speeds the appearance of new infections and the spread of old ones, and as degenerative diseases increasingly mingle with antimicrobial resistant pathogens in the same population, this model may help make sense of an increasingly complex disease-scape. In this way, epidemiologists may be able to use the model to anticipate future trends in the areas they study. For example, as the age of onset of chronic diseases is delayed in newly high-income countries experiencing the third epidemiological transition, it should be possible to plan for shifting disease burden by allocating more funds to research and health care provision for geriatric disorders. Similarly, by recognizing the distal causes that shape the epidemiological transitions, it should be possible to craft health policies that simultaneously address multiple outcomes. This could be achieved by manipulating the factors that underlie the overnutrition associated with the second epidemiological transition, for example, to reduce the incidence of diseases like diabetes, stroke, heart failure and cancer.

Like any model, this one is continuously being modified, and there is no doubt that it could benefit from greater attention from epidemiologists. The model rests on our understanding of the increase and decrease in different types of diseases. But dividing diseases into “types,” such as infectious *vs.* degenerative or human-made, is a difficult exercise. Many infectious diseases, for example, can to some extent be considered anthropogenic (e.g., hospital acquired infections). In addition, establishing whether there is an increase or decrease in the incidence of a disease is a difficult problem well known to epidemiologists, who are used to considering factors such as ascertainment bias. It has already been observed that difficulties in this area of study, including operationalizing terms and critically evaluating the evidence underlying the theory, could be profitably addressed by epidemiologists [16], and it will be interesting to see what changes and uses the future has in store for the epidemiological transition model.

## Figures and Tables

**Table 1.** The Three Epidemiological Transitions.

**Transition**	**Paleolithic Baseline**	**First Transition**	**Second Transition**	**Third Transition**
**Time Period**	Pre-Neolithic Cultures; More recent hunter-gatherer cultures with little outside contact	Neolithic cultures-Early Modern Times in Western Europe and United States; Still characterizes many low-income countries	Early Modern times to 20th century in Western Europe, United States; Occurred more recently in some other high-income countries and is in progress in lower income countries	End of the 20th century to the present, global
**Characteristics**	Pre-agricultural Low mortality and fertility rates Small population size Varied diet	Agricultural High mortality and fertility rates Large population size Diet heavily reliant on crops	Agricultural Low mortality and initially high then low fertility rates Large population size Increased life expectancy Varied diet, overnutrition common Discovery of antimicrobials and vaccines, improved hygiene	Agricultural Large population size Declining life expectancy? Failure of antimicrobials Rapid spread of novel infections Age of onset of chronic diseases delayed in high-income countries
**Common causes of morbidity and mortality**	Infections such as tapeworms, body lice, pinworms, typhoid, staph, and possibly yaws	Infections such as malaria, smallpox, measles, tuberculosis Nutritional deficiencies	Degenerative diseases such as heart failure, stroke, diabetes, cancer Allergies, asthma, autoimmune diseases Sexually transmitted infections such as HSV-2, gonorrhea, HIV	Those diseases present in the 2^nd^ transition Antibiotic resistant forms of tuberculosis, strep, staph, *etc*.
